# ACERTO Project - 15 years changing perioperative care in Brazil

**DOI:** 10.1590/0100-6991e-20202832

**Published:** 2021-01-13

**Authors:** JOSÉ EDUARDO DE-AGUILAR-NASCIMENTO, ALBERTO BICUDO SALOMÃO, CERVANTES CAPOROSSI, DIANA BORGES DOCK-NASCIMENTO, PEDRO EDER PORTARI-FILHO, ANTÔNIO CARLOS LIGOCKI CAMPOS, LUIZ EDUARDO IMBELLONI, JOÃO MANOEL SILVA-JR, DAN LINETZKY WAITZBERG, MARIA ISABEL TOULSON DAVISSON CORREIA

**Affiliations:** 1 - Centro Universitário de Várzea Grande (UNIVAG), Direção do Curso de Medicina - Várzea Grande - MT - Brasil; 2 - Universidade Federal de Mato Grosso, Curso de Pós-Graduação em Ciências da Saúde - Cuiabá - MT - Brasil; 3 - Universidade Federal de Mato Grosso, Departamento de Cirurgia - Cuiabá - MT - Brasil; 4 - Universidade Federal do Estado do Rio de Janeiro (UNIRIO), Departamento de Cirurgia Geral e Especializada - Rio de Janeiro - RJ - Brasil; 5 - Universidade Federal do Parana, Departamento de Cirurgia - Curitiba - PR - Brasil; 6 - Hospital das Clínicas Municipal, Anestesia - São Bernardo do Campo - SP - Brasil; 7 - Universidade de São Paulo, Divisão de Anestesiologia - São Paulo - SP - Brasil; 8 - Universidade de São Paulo, Departamento de Gastroenterologia - São Paulo - SP - Brasil; 9 - Universidade Federal de Minas Gerais, Departamento de Cirurgia - Belo Horizonte - MG - Brasil

**Keywords:** Preoperative Care, Postoperative Care, Nutrition Therapy, Postoperative Complications, Length of Stay, Cuidados Pré-Operatórios, Cuidados Pós-Operatórios, Terapia Nutricional, Complicações Pós-Operatórias, Tempo de Internação

## Abstract

The ACERTO project is a multimodal perioperative care protocol. Implemented in 2005, the project in the last 15 years has disseminated the idea of a modern perioperative care protocol, based on evidence and with interdisciplinary team work. Dozens of published studies, using the protocol, have shown benefits such as reduced hospital stay, postoperative complications and hospital costs. Disseminated in Brazil, the project is supported by the Brazilian College of Surgeons and the Brazilian Society of Parenteral and Enteral Nutrition, among others. This article compiles publications by the authors who belong to the CNPq research group “Acerto em Nutrição e Cirurgia”, refers to the experience of other national authors in various surgical specialties, and finally outlines the evolution of the ACERTO project in the timeline.

## RESUMO

O projeto ACERTO é um protocolo multimodal de cuidados perioperatórios. Implementado em 2005, o projeto, nos últimos 15 anos, tem disseminado a ideia de moderno protocolo de cuidados perioperatórios baseados em evidência e com atuação interprofissional. Dezenas de estudos publicados com o uso do protocolo têm mostrado benefícios como redução do tempo de internação, complicações pós-operatórias e custos hospitalares. Disseminado pelo Brasil, o projeto tem apoio do Colégio Brasileiro de Cirurgiões e da Sociedade Brasileira de Nutrição Parenteral e Enteral, entre outros. Este artigo compila publicações dos autores que compõem o grupo de pesquisa do CNPq “Acerto em Nutrição e Cirurgia”, cita a experiência de outros autores nacionais em diversas especialidades cirúrgica e finalmente, delineia a evolução do projeto ACERTO ao longo da linha do tempo.

## INTRODUCTION

The Nutrition and Surgery Research Group, from the Federal University of Mato Grosso, (UFMT) was structured at CNPq (National Council for Technological and Scientific Development / Brazil) at the end of the last century and, among other lines of research, aimed at increasing the amount of scientific information on perioperative care, an extremely new topic for the Brazilian reality at that time. Studies like “Implications of malnutrition in surgery”¹, “Early discharge in cholecystectomy”² and “Early feeding after intestinal anastomoses: risks or benefits?”³, published before the creation of ACERTO, formed the structure of the perioperative care protocol that would arise. Likewise, other publications involving nutritional therapy in surgery also contributed, notably evaluating malnutrition of the surgical patient¹, bacterial translocation^4^ ^,^ ^5^, nutritional therapy^6^, and use of probiotics^7^. 

In 2004, originally in the form of a research project, we created the project named “Acceleration of Full Postoperative Recovery” (ACERTO), thereafter known as the “ACERTO Project”. Using the “breakthrough” method, the ACERTO Project was implemented in the second half of 2005, after a six-month audit in the Surgery ward of the Julio Muller University Hospital (HUJM, Faculty of Medicine, UFMT). The references to the creation of ACERTO were the fast-track protocols^8^ ^,^ ^9^, originating in the eighties of the last century, as well as the European Enhanced Recovery After Surgery (ERAS)^10^. The once innovative concepts of fast-track were assimilated in the first studies of the group²^,^³. In the definition given by Kehlet & Wilmore, these protocols were the combination of various elective surgery perioperative care techniques: epidural or regional anesthesia, minimally invasive surgery, adequate pain control, postoperative aggressive rehabilitation, early oral/enteral intake, and early walking^11^. Likewise, the aforementioned ERAS program had a great influence, increasing the range of care recommended by the fast-track protocols and including the protocol of preoperative fasting abbreviation for only two hours and the offer of drinks containing carbohydrates^12^ ^,^ ^13^, although it was originally designed for use more specifically in colorectal surgery. ACERTO brought a new look to fast-track. In addition to having been based on the application in an epidemiological and social reality quite different from that of the protocols that preceded it (in Latin America), ACERTO brought a new vision for the application of multimodal conducts, extended not only to a single surgical specialty, but to a wide range of operative procedures with quite different clinical profiles, something common in the General Surgery wards of Brazilian public and private hospitals. In addition, the ACERTO Project showed that its adoption also benefited patients undergoing minor procedures, such as herniorrhaphies and cholecystectomies^14^ ^,^ ^15^. One of the notorious consequences of this is reflected in the participation, after some years of ACERTO in Brazil, by one of its creators (JEA-N), as co-author in the publication of the first ERAS guidelines, for a specific (non-colorectal) procedure, in this case, pancreaticoduodenectomy^16^.

### Planning and Implementation of the ACERTO Project

ACERTO was implemented in the HUJM with the “breakthrough” method. Succinctly, this method comprises four stages: (1) data collection on an initial audit, (2) preparation and drafting of the change plan, (3) collection of new data after implementation, and (4) analysis of new data and comparison with the initial collection^17^. After the analysis, the four-phase cycle is repeated, adjusting plans and reporting results. Thus, in the HUJM, the initial audit was carried out for six months, without the knowledge of the service’s professors and residents, seeking consistent information regarding preoperative fasting time, time until the beginning of postoperative intake, volume of perioperative fluids, clinical outcomes (postoperative morbidity), hospitalization time, and mortality. These data were presented in a full-day workshop, in July 2005, for the multidisciplinary team involved in perioperative care in the HUJM. In addition to displaying the findings, the goals of this meeting were also to 1) discuss the true statistics of what was happening in that surgical ward and 2) propose and discuss the new protocol (ACERTO). Also, for the first time we promoted the controversy between what was imaginary (thinking and affirming what I think I do, for example: I discharge patients on the 1st postoperative day) and what was reality (fact, evidence, as per example: mean hospitalization days for a given operation) in the service. It was impressive! Knowing that the time of preoperative fasting (believed to be between six and eight hours) was in average 16h (reaching 24h!), that the volume of crystalloids patients received perioperatively was high and quite different than imagined. These, among other important information on the reality observed by the audit raised those professionals’ awareness of the need for change. After that, the audit process continued, extending until December 2005, sufficient time so that differences were statistically demonstrated. Then came the first publication, which presented ACERTO to the national scientific scenario in the first half of 2006. It revealed important new information, such as the compliance to the conducts of the new Protocol, the impact on the improvement of results, such as decreased length of stay to two days, and reduction of the surgical site infection (SSI) rate^18^. These initial findings increased knowledge and acceptance of the protocol within the hospital, bringing anesthesiologists and surgeons closer. The result was the generation of even more consistent data, published in 2008^19^ and 2011^20^, respectively after three and five years of implementation. These three publications definitely solidified the ACERTO Project at the HUJM. The 2011 study involved approximately 5,000 patients and, for the first time, showed a reduction in postoperative mortality with the use of the ACERTO project^20^.

### Evolution of the ACERTO project

**Figure 1:**
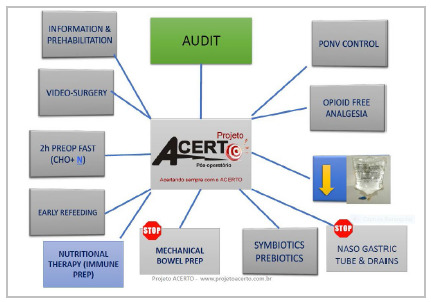
Perioperative care ACERTO protocol.

The evolution of perioperative care has been remarkable since the implementation of the ACERTO Project, in 2005. The protocol is dynamic and associates new evidence every year. To the initial protocol were added new recommendations over the years, such as pre-habilitation^21^ and use of probiotics and symbiotics^22^ ^,^ ^23^, always based on evidence from published works, both in Brazil and abroad. The relationship between nutritional risk and muscle function^24^ and preoperative sarcopenia with the likelihood of complications or postoperative mortality^25^ has been the focus of the recent work of the ACERTO group. The ACERTO Project currently has 12 recommendations (Figure 1). Recently, the project incorporated the idea of “ACERTO after discharge”. This need for a discharge plan and good guidance after discharge, especially in continuity with the nutritional therapy given at the hospital, aims to reduce hospital readmissions^26^ ^,^ ^27^. 

### Disseminating the ACERTO project

The group’s next step was to disseminate ACERTO to other hospitals in the city of Cuiabá^28^ and, later, to other hospitals in the country. This mission was initially assumed by the group of researchers who were at the head of the project since its inception (JEA-N, CC, ABS). These began to travel through various cities of the country, presenting at conferences and lecturing at major Medical Societies events, medical schools, Universities, Public and Private Hospitals, and even at the Ministry of Health. Importantly, the ACERTO has always been and still is disclosed in essence, a program first of all educational and non-profit, valuing accessibility to the exchange of information between the various services in Brazil and Latin America, and the joint search for the best evidence-based practices.

The Brazilian College of Surgeons (CBC) and the Brazilian Society of Enteral and Parenteral Nutrition (BRASPEN) were the first societies to approve and endorse ACERTO, something fundamental for the validation throughout the national territory. In 2009, one of the studies published by authors belonging to the original research group of the ACERTO Project was awarded the “Oscar Alves Award”, an important honor given to the best work published in the Journal of the Brazilian College of Surgeons the previous year^29^. Contributing to the spread of the necessity of the changes proposed by ACERTO in the Brazilian reality, the multicenter study BIGFAST, published in 2014, showed that the alarming preoperative fasting time found in HUJM in 2005 was not a local problem, but a national one. Data from 17 Brazilian hospitals including more than 4,000 patients showed that the preoperative fasting time in Brazil in all regions was very high and similar to that reported by the HUJM^30^. In 2017, twelve years after the original design, CBC and BRASPEN jointly published the ACERTO Directive of perioperative nutritional interventions^31^. Over time, new anesthesiology researchers also started to propagate the ACERTO publications^32^ ^,^ ^33^ and events (LEI, JSJ).

In this regard, other important Medical Societies, both belonging to the surgical setting as other areas, such as the Brazilian Society of Anesthesiology (SBA), as well as Hospitais and Research Groups, began to discuss the ACERTO project in their events. The theme “ACERTO” evolved to become part of education, both in undergraduate medicine and in medical residency, in various services and faculties in the country. Similarly, ACERTO content came to be present in other latu sensu and stricto sensu postgraduate modalities, becoming part of the literature for several medical selection processes, from specialist title tests to the recognition of foreign doctors’ degrees in Brazil.

### ACERTO Websites

To help spread the ACERTO Project, participants of the original group created two Internet websites, www.projetoacerto.com.br and www.periop.com.br (ABS), which, with thousands of hits over the years, offer literature and updates on modern perioperative care.

### Specialties within the ACERTO project

Since its inception, the use of the ACERTO protocol has accelerated the postoperative recovery of patients both undergoing medium-sized procedures^34^ ^,^ ^35^ and major ones, involving resections of the upper^36^ and lower^37^ ^,^ ^38^ gastrointestinal tract. All this, associated with several publications in abbreviation of preoperative fasting, including studies on safety and benefits^39-47^ and on perioperative intravenous fluid prescription^29^ ^,^ ^48^ ^,^ ^49^, increased the visibility of the ACERTO project. In fact, in any patient undergoing an operative procedure, prolonged fasting in the preoperative period decreases the functionality (strength) of the patients in the postoperative period^50^, increases insulin resistance^38^ and acute phase inflammation^51^. Thus, elderly^52^, oncological^18^, orthopedic^53^ ^,^ ^54^ patients, and those undergoing myocardial revascularization^55^ ^,^ ^56^ were subgroups of surgical patients in which the ACERTO Project has shown significant results improvements. Likewise, the adoption of the ACERTO Project was very safe and also promoted a reduction in hospital stay in patients undergoing bariatric surgery^57^ ^,^ ^58^. Recently, the dissemination of good data on the use of the protocol in pediatric surgery has attracted these specialists to ACERTO^59^ ^,^ ^60^.

### ACERTO Symposiums

The so-called “ACERTO Symposium” was first held in 2008 in the city of Cuiabá-MT, for the discussion of perioperative care in an interdisciplinary environment. From the following year on, the ACERTO Symposium became a national event, with support from the CBC, BRASPEN, and regional anesthesia societies. The increasing number of attendees led the event to capitals in the Brazilian Southeast Region, such as Rio de Janeiro (2014 and 2015) and São Paulo (from 2017 on), to attract more public and disseminate the idea. In repeating events, from 2010 on we had at the ACERTO symposium distinguished professors, both national and international, including members of the European group ERAS. Since 2017, the ACERTO symposium happens in São Paulo, with about 1000 participants. Latin American events from several countries (Mexico, Paraguay, Argentina, Peru, Colombia, Panama, Venezuela) have included ACERTO themes on their schedule. Since 2010, ACERTO has also been part of the scientific programming of the biannual World Congress of Surgery, and notably of IASMEN (International Association for Surgical Metabolism and Nutrition) as a society of the International Surgical Society (ISS). It is noteworthy that researchers and health professionals from Brazil and abroad recognize the ACERTO Project as the first multimodal protocol built and practiced in Latin America^61^ ^,^ ^62^. On the other hand, in 2011 the ERAS group organized the first International ERAS Congress in the city of Cannes (France), and a member of the ACERTO group (JEA-N) was included in that scientific program.

### The ACERTO Book

The ACERTO book had its first edition printed locally in 2009, but it was from the second edition, in 2011, that it gained national repercussion and became better known and disseminated. Bringing together a select group of authors involved with ACERTO, the book assists in the implementation and comprehensive discussion of all the evidence-based perioperative prescriptions that constitute the protocol. Given the changes in evidence that naturally occur with new data from the literature, in 2016 the third edition of the book was launched and, in 2020, a fourth edition became available. In the latter, there is an increase in chapters on anesthesiology, with the inclusion of three renowned specialists in this area, among the co-organizers of the book. This edition has 36 chapters, in contrast to only 16 in the first one.

### Publications in Brazil in the context of the ACERTO Project or ERAS protocol

After the first publication, in 2006, and with the creation of the CBC Perioperative Care Group, the number of studies on multimodal protocols grew in the country. Ludwig et al., in Southern Brazil, reported that ACERTO showed good results and that modifications of the perioperative care protocol should be encouraged, as they speed patient recovery^63^. Imbelloni et al. published several studies reporting good results in orthopedic and elderly patients with modifications to the traditional protocol, including abbreviated preoperative fasting, reduction of intravenous fluids, and blockage analgesia^32^ ^,^ ^64^ ^-^ ^66^. Ravanini et al. reported the use of drinks containing carbohydrates and whey protein two hours before anesthetic induction, improving postoperative insulin resistance in patients undergoing video cholecystectomy^67^. Marquini et al. found benefits in reducing nausea and vomiting with the abbreviation of fasting in gynecological surgery^68^. Lucchesi and Gadelha also found prolonged preoperative fasting, as well as the association of this factor with longer hospital stay^69^. Henriques and Correia showed that it is not necessary to prescribe crystalloid intravenous liquid in the immediate postoperative period of laparoscopic cholecystectomy^70^. Using the ERAS protocol, Teixeira et al. reported the preliminary results with 48 patients in 2019^71^. The Curitiba group, led by Campos (ACLC), demonstrated benefits of the perioperative use of pro and symbiotics in both colorectal cancer^22^ and bariatric^23^ surgery .

## FINAL CONSIDERATIONS

The ACERTO Project has 15 years of history and is definitely established in Brazil. However, it is still unknown by a large number of health professionals involved with perioperative care. The continuity of the efforts of the authors who are now members of the research group “Acerto em Nutrição e Cirurgia” (Acerto in Nutrition and Surgery), registered in the CNPq database, will certainly impact the expansion of research in perioperative care and in the use of the ACERTO protocol. An audacious goal of the group is the creation of a public policy on evidence-based perioperative care within the Brazilian Unified Health System (SUS). The future is promising!
